# Emerging Molecular Mechanisms in Malaria Pathogenesis and Novel Therapeutic Approaches: A Focus on *P. falciparum* Malaria

**DOI:** 10.3390/biom15071038

**Published:** 2025-07-17

**Authors:** Adekunle Sanyaolu, Aleksandra Marinkovic, Stephanie Prakash, Vyshnavy Balendra, Omar Shazley, Tatiana Gardellini, Abdul Jan, Kokab Younis, Chuku Okorie, Ricardo Izurieta

**Affiliations:** 1 Department of Biomedical Sciences, School of Health Professions, D’Youville University, Buffalo, NY 14201, USA; chukuokorie@yahoo.com; 2 School of Basic Medical Sciences, Saint James School of Medicine, Park Ridge, IL 60068, USA; marinkovica1@gmail.com (A.M.); stephaniexprakash@gmail.com (S.P.); veeb023@gmail.com (V.B.); omarshazley@gmail.com (O.S.);; 3 Office of Graduate Studies, Universidad Especializada de Las Américas UDELAS, Panama City 0849-0141, Panama; tatiana.gardellini@hotmail.com; 4 Faculty of Nursing, University of Calgary, Calgary, AB T2N 1N4, Canada; kokabyounis@gmail.com; 5 School of Public Health and Health Sciences, College of Health, Human Services and Nursing, California State University Dominguez Hills, Carson, CA 90747, USA; rizurieta@csudh.edu

**Keywords:** malaria, treatment, vaccine, elimination, omics

## Abstract

Malaria is still one of the biggest global health problems, especially in parts of the world, such as sub-Saharan Africa, which remains most heavily affected. Despite significant advancements in testing, treatment, and prevention, malaria continues to seriously impact millions, primarily young children and populations in rural and impoverished areas. This paper looks at how the malaria parasite works inside the body, how it avoids the immune system, and how it becomes resistant to current drugs. Thanks to new advances in genetic and biochemical research, scientists are discovering new weak points in the parasite that could lead to better treatments. New vaccines, like RTS, S and R21, along with antibody-based therapies, offer renewed hope; however, extending the duration of the immunity they induce and ensuring effectiveness across diverse parasite strains remain significant challenges. Solving the malaria crisis will require more than science—it also necessitates equitable and timely access to treatments, robust health systems, and international collaboration. Continued research and global cooperation bring the world closer to ending malaria for good.

## 1. Introduction

Malaria, also known as paludism, is an acute febrile disease caused by parasites of the genus *Plasmodium* that is transmitted to humans through the bite of a female mosquito of the genus *Anopheles* [1]. The disease causes an estimated 619,000 deaths annually [2].

Although malaria is a preventable and treatable disease, it remains a global public health problem with high mortality and morbidity rates [3]. Many cases of malaria deaths occur in Africa, most of whom are children [4]. According to the 2023 World Malaria Report, globally, 249 million cases of malaria were recorded in 85 endemic countries in 2022 [5]. However, in the Americas, a significant decrease in cases was observed, from 1.5 million in 2000 to only 0.6 million in 2022 [5]. This decline is attributed to increased diagnosis and treatment, as well as to lower population mobility due to the emergence of COVID-19 [5]. Globally, malaria deaths also decreased from 864,000 in 2000 to 576,000 in 2019 [5]. Between 2000 and 2022, the WHO Region of the Americas achieved a 63% reduction in malaria deaths, from 850 to 343. The mortality rate also fell by 71%, from 0.7 to 0.2 per 100,000 people at risk [5].

Malaria is a protozoan disease transmitted by *Anopheles* mosquitoes. Five species of the genus *Plasmodium* cause all malarial infections in human beings. Among the numerous species within the genus *Plasmodium*, five are known to cause malaria in humans: *Plasmodium falciparum*, *Plasmodium vivax*, *Plasmodium ovale*, *Plasmodium malariae*, and *Plasmodium knowlesi*. Each species varies in geographical distribution, clinical presentation, and potential severity, with *P. falciparum* being the most lethal and prevalent in sub-Saharan Africa [6,7,8]. After transmission from an infected mosquito, the parasite initially develops in the liver, subsequently invading red blood cells where it multiplies, evades immune responses, and induces clinical symptoms. Malaria parasites invade red blood cells (RBCs) and undergo complex developmental stages that lead to cycles of fever and contribute to disease severity through immune evasion, sequestration, and the destruction of host cells. Among these, *Plasmodium falciparum* is particularly associated with the most severe clinical outcomes.

In cases of malaria, common clinical manifestations include anemia, metabolic acidosis, hypoglycemia, pulmonary edema, acute kidney injury (AKI), and jaundice, reflecting the systemic and potentially severe impact of the disease on multiple organ systems. However, it is important to note that clinical presentations can vary significantly, depending on the infecting *Plasmodium* species [6]. In Global Technical Strategy for Malaria 2016–2030, a key goal of the World Health Organization (WHO) is to reduce malaria incidence and mortality by 90% between 2016 and 2030, and to eliminate malaria in 35 countries, ensuring no resurgence in countries where it has been eliminated [7,8].

Malaria prevention strategies include vaccination, vector control measures, such as insecticide-treated bed nets and indoor spraying, as well as chemoprophylaxis and chemoprevention using antimalarial medications in high-risk populations [6]. The RTS,S subunit vaccine (R = repeat region of the circumsporozoite protein [CSP], T = T-cell epitope of CSP, S = surface antigen of hepatitis B virus [HBsAg], S [extra] = added HBsAg to help form virus-like particles for enhanced immune response), which targets the circumsporozoite protein of *Plasmodium falciparum* and is formulated with the potent AS01 adjuvant (A = adjuvant system, S01 = specific formulation in the adjuvant system series), is the first malaria vaccine to be approved by the WHO and is currently being rolled out in several African countries [6]. In addition to the advancement of new malaria vaccines, there have been recent improvements in the development of monoclonal antibodies (mAbs) for the prevention of malaria. Monoclonal antibodies are laboratory-produced molecules that can mirror the immune system’s ability to fight off harmful pathogens (viruses, bacteria, yeasts, and parasites) [8].

Timely diagnosis has also played a critical role in malaria control. While rapid diagnostic tests (RDTs) and microscopy remain commonly used tools, their sensitivity and accessibility in rural and underserved areas remain inconsistent [9]. There has been significant progress in malaria control in the last two decades, with a decline in mortality and morbidity. The control of malaria vectors in Africa and other malaria-endemic countries has received increased attention in the past decade, with the scaling up of insecticide-treated bed nets and indoor residual house spraying with insecticides [9]. *Anopheles gambiae* complex is considered one of the most efficient malaria vectors worldwide, with its highest impact observed in sub-Saharan Africa [10]. Insecticide-treated bed nets (ITNs) and indoor residual spraying (IRS) have been broadly used as front-line tools against malaria vectors. Chemical insecticides remain the most widely used form of mosquito control. With rising mosquito resistance, the effectiveness of these methods is increasingly compromised, highlighting the urgent need for alternative or integrated vector management strategies [11]. However, the insecticide resistance of malaria vectors is threatening these vector control interventions.

The increased use of insecticides in malaria control programs has significantly raised resistance levels in many countries, particularly against pyrethroids, potentially undermining the effectiveness of vector-control measures [12]. With rising drug resistance and global climate shifts contributing to potential resurgence in previously controlled regions, understanding and addressing these biological complexities is imperative. Furthermore, bridging scientific discovery with policy, healthcare infrastructure, and global equity efforts is essential for long-term, sustainable malaria eradication.

Malaria elimination has also been affected by geographical factors, such as altitude, temperature, and humidity, as well as rainfall patterns, proximity to water bodies, land use, and vector distribution [13]. Sociodemographic characteristics include difficult access to malaria treatment and the implementation of vector-control measures [13]. There are also sociocultural aspects such as the mobility of people, the degree of acceptance of preventive measures in vulnerable populations, and the challenges of ensuring the timely administration of antimalarial treatment [13]. Another major challenge is the resistance of the parasite to drugs and the resistance of mosquitoes to treatment, as noted in the Global Technical Strategy for Malaria 2016–2030 [14].

In some areas, despite substantial financial investment in malaria control, commensurate reductions in case numbers have not been noted. It may be that the epidemiology of malaria in these areas remains underestimated. In regions with low overall transmission, small pockets of stable transmission can serve as reservoirs for ongoing spread. In addition, the prevalence of asymptomatic malaria is often underestimated. Counterfeit and inadequate drugs are a major threat to malaria control; more active countermeasures, stronger legislation, and more thorough investigations are needed [6].

This paper aims to explore the molecular mechanisms underlying malaria pathogenesis and review recent advances in therapeutics. By examining how the parasite operates at a cellular and molecular level, and evaluating the most promising therapeutic strategies in development, we seek to highlight critical opportunities for innovation in malaria control.

## 2. Emerging Molecular Mechanisms in Malaria Pathogenesis

### 2.1. Molecular Mechanisms

Malaria pathogenesis involves complex interactions between the *Plasmodium* parasite and its human host, characterized by distinct life cycle stages and molecular mechanisms that contribute to disease severity and drug resistance. Understanding these processes is crucial for developing effective treatments and preventive measures.

**Figure 1:**
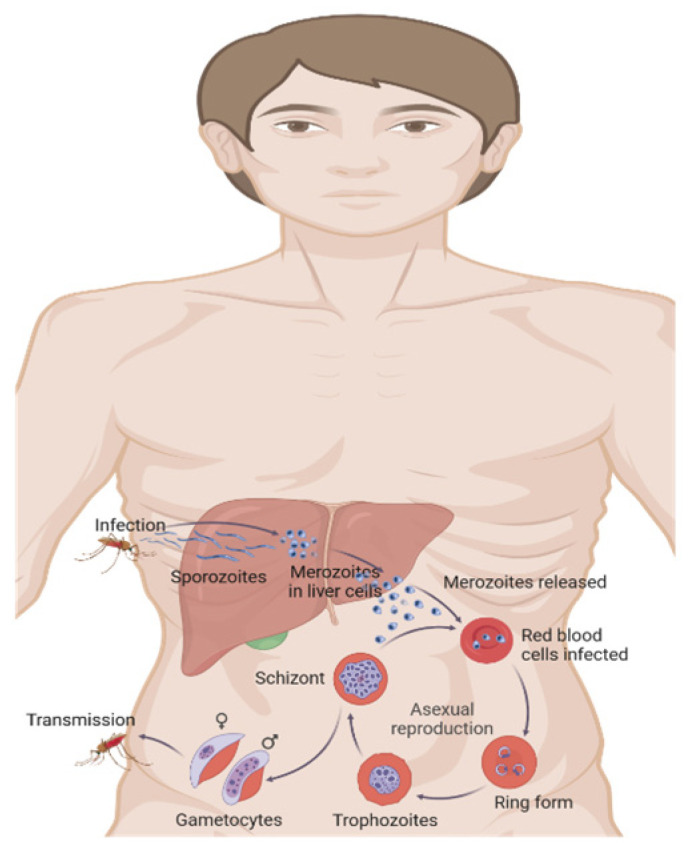
Malaria life cycle. Created in Biorender by Ricardo Izurieta (2025) https://app.biorender.com/illustrations/65e5f863bef1585d94d47108 (accessed on 26 June 2025).

*Plasmodium* is a parasite with a complex life cycle (Figure 1) that alternates between human hosts and mosquito vectors. The process of infection starts when a female Anopheles mosquito, carrying the parasite, bites a person and releases sporozoites into their bloodstream [15]. Sporozoites migrate rapidly to the liver, infecting hepatocytes and differentiating into merozoites [16]. Crucially, in *Plasmodium vivax* and *Plasmodium ovale* infections, some sporozoites form dormant stages called hypnozoites, which can persist in the liver and reactivate weeks, months, or even years later, leading to relapse episodes [17]. Recent molecular studies have elucidated that hypnozoite formation and persistence involve complex genetic and epigenetic regulatory mechanisms, with evidence pointing towards specific transcription factors and histone modifications that maintain dormancy [17,18].

After hepatic maturation, merozoites are released into the bloodstream, invading erythrocytes and initiating the erythrocytic cycle [16]. Within erythrocytes, merozoites develop into trophozoites, which multiply asexually, subsequently, rupturing the cell and releasing new merozoites. Some merozoites differentiate into gametocytes, which are ingested by another mosquito during feeding, thus completing the cycle [15].

Severe malaria caused by *Plasmodium vivax* has gained increasing recognition due to its significant morbidity [19]. Unlike the earlier notion of *P. vivax* causing mild infections, recent studies have identified severe manifestations, including anemia, acute respiratory distress syndrome (ARDS), thrombocytopenia, and renal impairment [19]. Molecular mechanisms underlying severe *P. vivax* infections are associated with heightened inflammatory responses, excessive cytokine release, and dysregulated immune responses triggered by parasite-specific proteins such as *Plasmodium vivax* Duffy Binding Protein (PvDBP) and *Plasmodium vivax* Apical Membrane Antigen 1 (PvAMA1), which facilitate parasite invasion and immune evasion [19,20].

Similarly, *Plasmodium knowlesi*, primarily a simian parasite infecting macaques, has emerged as a significant zoonotic cause of severe malaria in Southeast Asia [21]. Severe *P. knowlesi* infections can rapidly progress to multi-organ dysfunction, characterized by acute kidney injury, severe respiratory distress, and metabolic acidosis [21]. Recent genomic and transcriptomic analyses have revealed that severe *P. knowlesi* infection involves distinct parasite–host interactions, notably increased expression of genes encoding erythrocyte-binding proteins (EBPs) and variant antigens, which facilitate robust invasion and rapid multiplication within erythrocytes, leading to high parasite biomass and profound inflammation [21,22].

### 2.2. Host–Pathogen Interaction

*Plasmodium* parasites invade host cells using specialized organelles such as micronemes, rhoptries, and dense granules. Parasite surface proteins, including merozoite surface protein 1 (MSP-1) and apical membrane antigen 1 (AMA-1), facilitate the attachment to, and invasion of, erythrocytes [23]. Rhoptry proteins assist in creating the parasitophorous vacuole, which is crucial for parasite entry and protection from host immune responses [24].

**Figure 2:**
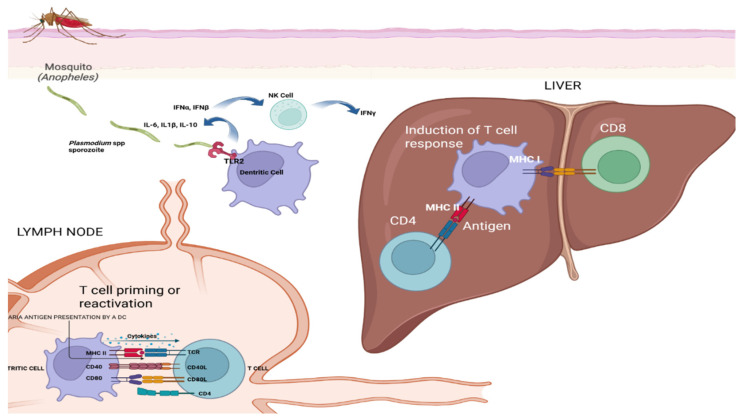
Immune response to malaria infection. An illustration detailing the interactions between *Plasmodium* sporozoites and the human immune system, highlighting key events such as antigen presentation, T-cell activation, and cytokine-mediated response. Created in Biorender by Ricardo Izurieta (2025) https://app.biorender.com/illustrations/6830a57a1a64c6d0c5eff1ff (accessed on 26 June 2025).

The immune system responds to *Plasmodium* infection through both antibody-mediated and cell-based mechanisms (Figure 2). Antibodies directed against proteins on the surface of merozoites can interfere with the parasite’s ability to invade red blood cells [23]. Nonetheless, *Plasmodium* has evolved strategies to escape immune detection, including the ability to alter the expression of *Plasmodium falciparum* erythrocyte membrane protein 1 (PfEMP1), which is displayed on the surface of infected red blood cells, thereby reducing the effectiveness of immune clearance [25].

The image in Figure 2 illustrates the immune response to malaria infection, detailing the journey and interaction of *Plasmodium* sporozoites within the human body and antigen presentation and T cell activation in malaria immunity, as follows:

Mosquito bite and sporozoite injection: An *Anopheles* mosquito bites human skin, injecting *Plasmodium* sporozoites into the bloodstream;

Dendritic cell capture: The sporozoites are captured by dendritic cells, which migrate to the lymph nodes;

Antigen presentation: In the lymph nodes, dendritic cells present *Plasmodium* antigens to T cells, initiating the immune response;

Interaction with NK cells: Dendritic cells interact with natural killer (NK) cells, releasing cytokines such as IFNγ and IL-12, which enhance the immune response;

T Cell priming and reactivation: The lymph node section shows T cell priming or reactivation, involving cytokines IL-12 and IL-10. CD4 and CD8 T cells interact with MHC molecules on antigen-presenting cells;

Liver stage: CD8 T cells recognize infected hepatocytes in the liver through MHC I-antigen complexes, leading to the destruction of infected cells;

Cytokine release: Various cytokines are released during these interactions, including IL-12, IL-10, and IFNγ, which help to modulate the immune response.

### 2.3. Molecular Mechanisms of Disease Severity

Malaria virulence primarily depends on parasite proteins essential for erythrocyte invasion and adherence to vascular endothelium. PfEMP1 variants facilitate the adhesion of infected erythrocytes to vascular endothelium, significantly contributing to severe pathology such as cerebral malaria [25]. Host genetic variations, notably sickle-cell trait (hemoglobin S-HbS) and glucose-6-phosphate dehydrogenase (G6PD) deficiency, affect malaria susceptibility and disease outcomes by altering erythrocyte physiology and inhibiting parasite growth [26]. Alterations in red blood cell (RBC) properties, including membrane rigidity and receptor density, significantly influence malaria pathogenesis. Parasite-induced modifications cause hemolysis, anemia, and impaired microcirculation, contributing to clinical manifestations like severe anemia and cerebral malaria [16].

Oxidative stress plays a significant role in malaria pathogenesis, characterized by an imbalance between reactive oxygen species (ROS) production and antioxidant defense mechanisms. Elevated ROS levels can damage erythrocyte membranes, leading to increased hemolysis and contributing significantly to anemia and impaired microcirculation [27,28]. Moreover, oxidative stress modulates immune responses, influencing disease severity and progression [27,28].

Lipid peroxidation results from excessive ROS and significantly damages erythrocyte membranes, compromising their integrity and function. This process exacerbates hemolysis, enhances inflammation, and contributes to the immunosuppression seen in severe malaria cases [28,29,30]. Lipid peroxidation by-products, such as malondialdehyde (MDA) and 4-hydroxynonenal (4-HNE), also act as signaling molecules, modulating inflammatory responses and contributing to malaria-induced pathology [30].

Malarial products, like hemozoin, a pigment produced during hemoglobin digestion by parasites, directly trigger inflammatory responses and oxidative stress in host tissues. Hemozoin accumulation exacerbates inflammation, immune dysregulation, and contributes significantly to the pathology of severe malaria [31]. Additionally, 4-hydroxynonenal (4-HNE), a lipid peroxidation by-product, can further aggravate oxidative stress and inflammatory responses through the modification of host proteins [29,30].

Post-translational modifications (PTMs) are crucial in regulating protein function during malaria infections. PTMs, such as phosphorylation, ubiquitination, and carbonylation, can alter erythrocyte and immune cell functions, exacerbating anemia and immunosuppression [32,33]. Recent proteomic and multi-omics studies have identified extensive PTMs in malaria-infected erythrocytes, suggesting their roles in modulating host–parasite interactions, protein stability, and the signaling pathways critical for disease progression [32,33].

### 2.4. Molecular Basis of Drug Resistance

Drug resistance in *Plasmodium*, particularly *P. falciparum*, arises from specific genetic mutations. Resistance to Chloroquine resistance primarily arises from mutations in the Plasmodium falciparum chloroquine resistance transporter (PfCRT) gene, while mutations in the Kelch13 (K13, Kelch propeller domain protein on chromosome 13) propeller domain have been linked to artemisinin resistance [34]. Chloroquine resistance involves reduced drug accumulation within the parasite’s digestive vacuole, due to mutated PfCRT transporters. Artemisinin resistance, characterized by delayed parasite clearance, typically involves mutations in K13, affecting parasite proteostasis pathways [34]. Transporter proteins, including PfCRT and *Plasmodium falciparum* multidrug resistance protein 1 (PfMDR1), play pivotal roles in drug resistance by actively transporting drugs away from their intracellular targets, thereby diminishing drug efficacy. Genetic mutations alter transporter function, reducing drug accumulation within the parasite’s compartments [35]. As the parasite continues to evolve under drug pressure, it is increasingly important to identify how genetic changes confer resistance and impact treatment efficacy. Investigating the genetic basis of resistance complements the broader systems-level insights provided by omics (proteomics, transcriptomics, genomics, and metabolomics) technologies, thereby enabling more comprehensive approaches to surveillance and drug design [36,37,38,39].

### 2.5. Recent Advances in Malaria Research

Malaria remains a significant global health challenge, with *Plasmodium falciparum* being the most virulent parasite responsible for the most severe cases. Recent scientific advances leveraging cutting-edge technologies have significantly improved the understanding of malaria, facilitating novel therapeutic and preventive strategies.

#### 2.5.1. Genomic and Transcriptomic Insights

Advances in next-generation sequencing (NGS) have revolutionized malaria research by offering detailed views of the genome and gene expression patterns of *Plasmodium* species. Through the whole-genome sequencing (WGS) of *P. falciparum* samples from various regions, researchers have uncovered significant genetic variations, including single-nucleotide polymorphisms (SNPs), structural rearrangements, and copy number changes, that play roles in both drug resistance and parasite virulence [40,41]. These genomic insights are critical for monitoring the emergence and spread of resistant strains and guiding targeted public health interventions.

Transcriptomic analyses using RNA sequencing have offered crucial insights into stage-specific gene expression during the parasite’s complex life cycle. Studies have identified coordinated transcriptional programs that govern transitions between liver, blood, and mosquito stages, as well as the differential expression of variant surface antigens such as var, rifin, and stevor gene families [42,43]. Moreover, single-cell transcriptomics has emerged as a powerful tool with which to uncover cellular heterogeneity among parasites, thereby elucidating mechanisms of phenotypic plasticity and immune evasion. The integration of transcriptomic and epigenomic datasets has further enhanced the understanding of how transcription factors, histone modifications, and non-coding RNAs regulate gene expression in response to environmental cues [44].

Importantly, genomic approaches have led to the identification of several novel drug targets. These include enzymes essential for parasite survival, such as dihydroorotate dehydrogenase (DHODH), a key component in pyrimidine biosynthesis [45], and apicoplast-targeted isoprenoid biosynthesis enzymes like IspD and IspF [46]. Additionally, transporter proteins such as PfATP4 (a sodium pump, *Plasmodium falciparum* ATPase 4) [47] and PfNCR1 (involved in lipid homeostasis) [48] have been validated as high-value antimalarial targets through genome-wide mutagenesis and chemical-genomics screens. These discoveries are guiding the development of next-generation therapeutics aimed at less mutable and more essential parasite pathways.

#### 2.5.2. Proteomics and Metabolomics in Malaria

Proteomic research has elucidated numerous parasite proteins essential for survival and host-cell invasion, significantly advancing our understanding of the invasion mechanisms employed by merozoites. Advanced proteomic techniques, particularly mass spectrometry, have enabled the identification and characterization of novel proteins that may serve as viable drug targets [49,50]. Among the novel proteins identified are rhoptry-associated proteins (RAPs), reticulocyte binding protein homologs (PfRh family), and members of the serine repeat antigen (SERA) family. These proteins are involved in red blood cell invasion, intracellular development, and immune modulation. In summary, proteomics focuses on understanding the parasite’s proteins and their roles in various biological processes [51].

Numerous post-translational modifications (PTMs) have been reported in malaria, significantly influencing parasite biology and host interactions. Parasite protein PTMs, such as phosphorylation, acetylation, methylation, and protein lipoxidation, play crucial roles in regulating invasion, growth, and immune evasion [52,53]. Phosphorylation has been extensively documented, particularly in the proteins involved in merozoite invasion and egress from infected erythrocytes, modulating their activity and interactions with host cells [54,55]. Acetylation and methylation of histones and transcription factors within the parasite regulate gene expression critical for life cycle progression and environmental adaptation [56,57]. Protein lipoxidation, resulting from oxidative stress, modifies parasite proteins, affecting their function and stability, thereby influencing parasite survival and pathogenicity [58].

Modifications of host proteins in infected red blood cells and host immune and epithelial cells have distinct implications for malaria pathogenesis. Within infected erythrocytes, host protein PTMs, such as phosphorylation and ubiquitination, alter membrane rigidity, signaling pathways, and antigen presentation, contributing to immune evasion and parasite persistence [59,60]. Lipoxidation of host erythrocyte membrane proteins due to parasite-induced oxidative stress contributes significantly to erythrocyte damage, hemolysis, and anemia [58]. In immune cells, PTMs, including phosphorylation and acetylation, regulate inflammatory responses, cytokine production, and apoptosis, shaping the host’s immune response during malaria infection [61,62]. PTMs in endothelial and epithelial cells further influence inflammation, barrier integrity, and tissue damage, exacerbating severe malaria manifestations such as cerebral malaria and acute lung injury [63,64].

At the same time, metabolomic research has shed light on the biochemical networks essential for *Plasmodium* survival, uncovering potential points of therapeutic intervention. Key metabolic processes, such as glycolysis, the pentose phosphate pathway (PPP), nucleotide synthesis, and mitochondrial energy production, have been identified as crucial for parasite viability. Notably, enzymes, including hexokinase, pyruvate kinase, glucose-6-phosphate dehydrogenase (G6PD), dihydrofolate reductase (DHFR), and the cytochrome bc1 complex, have been highlighted as promising drug targets [65,66]. Disrupting the functions of these enzymes can compromise ATP generation, nucleotide availability, redox balance, and iron utilization in the parasite.

Furthermore, metabolic processes, like fatty acid and lipid biosynthesis (e.g., FAS-II), heme detoxification (via hemozoin synthase), redox balance (via glutathione and peroxiredoxins), and protein turnover (ubiquitin-proteasome pathway), are essential for the parasite’s replication and survival. Inhibitors targeting these pathways, such as proteasome inhibitors or agents disrupting heme polymerization, have shown promise in preclinical studies [67,68]. Collectively, these insights into *Plasmodium* metabolism underscore the potential of targeting biochemical pathways unique to the parasite, paving the way for novel antimalarial therapies that complement traditional drug strategies.

#### 2.5.3. Understanding Antigenic Variation and Immune Evasion

Proteomic research has elucidated numerous parasite proteins essential for survival and host-cell invasion, significantly advancing our understanding of the invasion mechanisms employed by merozoites. Advanced proteomic techniques, particularly mass spectrometry, have enabled the identification and characterization of novel proteins that may serve as viable drug targets [69,70]. Among the novel proteins identified are rhoptry-associated proteins (RAPs), reticulocyte binding protein homologs (PfRh family), and members of the serine repeat antigen (SERA) family. These proteins are involved in red blood cell invasion, intracellular development, and immune modulation. In summary, proteomics focuses on understanding the parasite’s proteins and their roles in various biological processes [37].

A major challenge in malaria control is antigenic variation, predominantly driven by PfEMP1. PfEMP1 facilitates immune evasion through cytoadhering to host cells, enabling parasites to evade clearance by the immune system. Recent genomic and transcriptomic studies have elucidated the regulatory mechanisms underpinning PfEMP1 gene switching, enhancing our understanding of how parasites sustain chronic infections by continually altering their antigenic profile [71,72].

Insights into these immune evasion strategies have crucial implications for malaria vaccine development. Because PfEMP1 proteins are highly variable, immune responses against them tend to be strain-specific and short-lived. However, conserved regions within the PfEMP1 family—and other antigenic proteins—are now being investigated as targets for broadly protective vaccines. Additionally, understanding the epigenetic and transcriptional regulation of variant gene expression may open avenues for strategies that block antigenic switching altogether. Such approaches could enhance the immune system’s ability to recognize and eliminate the parasite, offering the potential for vaccines that generate long-lasting and cross-protective immunity [73].

Host immunosuppression represents an additional layer of immune evasion in malaria. Parasite-derived products, such as hemozoin and 4-hydroxynonenal (4-HNE), significantly alter host immune functions by modulating the expression of crucial immune receptors. For instance, hemozoin reduces the expression of receptors, such as CCR2, TNFR1/2, and MHC class II, impairing host-cell signaling pathways and cytokine responses critical for efficient parasite clearance [74,75]. Similarly, 4-HNE interferes with antigen presentation mechanisms and T-cell activation by modifying immune signaling proteins, further contributing to the immunosuppressive environment observed during malaria infection [30]. Targeting these immunosuppressive mechanisms could enhance the efficacy of vaccines and immunotherapies aimed at strengthening the host’s immune response against malaria.

While progress in understanding the molecular mechanisms of malaria pathogenesis has been substantial, translating these discoveries into effective treatments remains a critical priority. The spread of drug-resistant *Plasmodium falciparum* has created a pressing need for innovative therapies.

## 3. Emerging Therapeutic Strategies

Malaria control relies not only on therapeutic interventions but also heavily on preventative measures aimed at reducing transmission and infection rates. Vector control remains a cornerstone of prevention, achieved through insecticide-treated nets (ITNs), indoor and outdoor insecticide sprays, and antimalarial prophylaxis; however, insecticide resistance is an emerging challenge [76]. Significant progress has also been made in malaria vaccine development. For example, immunization with radiation-attenuated *Plasmodium falciparum* sporozoites (PfSPZ Vaccine) has shown high efficacy in controlled human malaria infection (CHMI) studies, demonstrating 89–100% protection [77]. Nearly 89% protection was observed after a single immunization targeting late liver-stage parasites, while a vaccine targeting earlier liver stages showed 95% efficacy [78]. Additionally, RH5.1, formulated with the Matrix-M adjuvant, is a soluble protein vaccine currently undergoing clinical trials as a candidate for blood-stage *P. falciparum* [79].

### 3.1. Novel Antimalarial Drugs

CYP450 enzymes metabolize drugs, often converting them into inactive forms. The metabolism of antimalarial drugs is highly dependent on cytochrome P450 (CYP450) enzymes, which impact the potency, efficacy, and safety. For instance, CYP2D6 (cytochrome P450 2D6) is required to activate primaquine; variations in this enzyme can impair drug activation and reduce efficacy against sporozoites [80]. Similarly, individuals carrying the alleles CYP2C8*2 (Cytochrome P450 2C8) have lowered enzymatic activity, compromising the efficacy of amodiaquine, which relies on CYP2C8 to convert into its active metabolite, desethyl-amodiaquine [81]. These genetic variations of CYP450 enzymes are therefore critical determinants of antimalarial treatment effectiveness and resistance management. Genetic differences in CYP450 enzymes can lead to variations in drug metabolism among individuals. Beyond host metabolism, recent research has increasingly focused on targeting the parasite itself through novel therapeutic approaches.

Epigenetic inhibitors, or “epidrugs,” are agents that are aimed at the parasite’s DNA enzymes, which include histone deacetylase and methyltransferase, and DNA methyltransferase. These treatment options inhibit the *Plasmodium falciparum* at numerous life cycle stages. Studies indicate that many of these inhibitors have been shown to be effective against gametocytes at low concentrations [82]. This novel therapeutic alternative has been able to treat artemisinin-resistant strains and combat drug resistance, due to its multistage activity [83].

Recent advances have also yielded new antimalarial compounds designed to overcome growing drug resistance in *Plasmodium* species [84]. Compounds, such as cipargamin (KAE609) and ganaplacide (KAF156), have been identified to target unique parasite pathways, offering alternative mechanisms to traditional pharmacological therapies [85]. These drugs exhibit activity against multiple life cycle stages of the parasite, including liver, asexual blood, and sexual forms, providing a comprehensive approach to treatment.

#### 3.1.1. Mechanism of Action of New Drug Candidates

Understanding the mechanisms of action for these new drug candidates is essential for their effective application in clinical settings. For instance, cipargamin targets the parasite’s ATP4 ion pump, disrupting sodium homeostasis [85,86], while ganaplacide [KAF156] interferes with protein trafficking and expansion of the endoplasmic reticulum [87]. These novel targets differ from those of existing drugs, reducing the likelihood of cross-resistance and enhancing the potential for successful treatment outcomes.

#### 3.1.2. The Role of Synthetic Chemistry and Natural Products in Drug Discovery

The role of synthetic chemistry and natural products in antimalarial drug discovery remains pivotal [88]. Natural compounds, like quinine and artemisinin, have historically been the cornerstone of malaria treatment [89]. Synthetic chemistry advancements have allowed for the modification of these natural products to improve their efficacy and pharmacokinetic properties [88]. In parallel, high-throughput screening of synthetic libraries has yielded novel compounds with potent antimalarial activity.

Building on the success of natural product-derived therapies, researchers have also turned to marine organisms for new leads. Plakortin, a natural compound isolated from the marine sponge *Plakortis simplex*, has demonstrated strong antimalarial activity. Its mechanism of action resembles that of artemisinin, relying on peroxide-dependent processes—specifically, lipid peroxidation—to disrupt parasite function [90]. Plakortin inhibits key enzymatic activity within the parasite, impairing its growth and maturation. In vitro studies have shown efficacy against both chloroquine-sensitive and -resistant *P. falciparum* strains. Notably, when combined with conventional treatments, like chloroquine, plakortin has shown enhanced therapeutic potential [91].

#### 3.1.3. Targeting the Malaria Life Cycle

Targeting the malaria life cycle at various stages is a strategic approach to disease control. While many antimalarial drugs focus on the asexual blood stages responsible for clinical symptoms, there is a growing emphasis on developing agents that target liver-stage parasites [92]. These interventions can prevent disease onset and interrupt transmission, thereby contributing to broader eradication efforts.

##### Drugs Targeting Specific Stages of the *Plasmodium* Life Cycle

Targeting specific stages of the *Plasmodium* life cycle is essential for comprehensive malaria control. Each developmental phase presents unique vulnerabilities; stage-specific therapies can enhance treatment effectiveness and limit transmission.

For instance, *Plasmodium vivax* hypnozoites represent a dormant liver-stage form responsible for relapses. Primaquine and tafenoquine are currently the only approved drugs that can eliminate these latent forms. However, both agents pose a risk of hemolytic anemia in patients with glucose-6-phosphate dehydrogenase (G6PD) deficiency, necessitating genotype-guided dosing to ensure both safety and efficacy [93].

Another important stage-specific target is hemozoin, a crystalline by-product formed when the parasite digests host hemoglobin within its acidic digestive vacuole. During this process, the toxic free heme is converted into insoluble β-hematin crystals [94]. When infected erythrocytes rupture, hemozoin is released into the bloodstream, where it is engulfed by macrophages and monocytes. This triggers an inflammatory response characterized by increased IL-1β levels, which impairs antigen presentation and induces lipid peroxidation [95]. Consequently, this process suppresses erythropoiesis and weakens immune function, contributing to malaria-related immunosuppression [96].

Drugs, like primaquine, are effective not only against liver-stage hypnozoites but also some gametocyte forms, reducing the chance of transmission [97,98]. Tafenoquine, meanwhile, offers a dual-action effect by targeting both liver-stage and blood-stage schizonts of *P. vivax*, making it particularly useful in relapse prevention and acute treatment [98,99].

Efforts are also underway to target sporozoites, the parasite form transmitted by mosquitoes before it infects the liver. Monoclonal antibodies, such as CIS43LS and L9LS, have demonstrated promising results in both animal models and controlled human malaria infection (CHMI) studies, effectively neutralizing the sporozoite surface antigen and offering pre-erythrocytic protection [100]. Additional monoclonal antibodies targeting cryptic epitopes, like pGlu-CSP, have shown enhanced protection when combined with vaccines [101].

Pre-erythrocytic vaccines, such as RTS, S, and R21, which also target the circumsporozoite protein (CSP), have reached advanced stages of clinical development. These vaccines have demonstrated up to 75% efficacy in Phase III trials, offering a strong preventative approach against initial infection [102]. Together, these therapies—whether prophylactic or curative—illustrate the growing precision in malaria drug development and the strategic importance of attacking the parasite at multiple stages of its complex life cycle.

##### Inhibiting Parasite Entry and Replication

Inhibiting parasite entry and replication within red blood cells is another focal point of antimalarial strategies [103]. Compounds that disrupt the interaction between merozoites and erythrocyte receptors through merozoite surface proteins (MSPs), like MSP1 and RON2, can effectively prevent invasion and subsequent replication [104,105]. Research in molecular mechanisms of parasite entry has identified potential targets for intervention, offering new avenues for therapeutic development.

### 3.2. Immunotherapies and Vaccines

#### 3.2.1. Recent Advancements in Malaria Vaccines (e.g., RTS,S/AS01)

The RTS, S/AS01 vaccine targets the circumsporozoite protein (CSP), which is a surface protein from the sporozoite stage of malaria parasites [106]. RTS, S is a recombinant vaccine that interacts with CSP, while the AS01 adjuvant portion of the vaccine induces the innate CD4+ T cell-mediated immune response [107,108]. Clinical trials have shown a decrease of 39% in cases and 29% of severe malaria cases in children between the ages of 5–17 months after the initial dose over a four-year follow-up duration [109]. The R21/Matrix-M vaccine has also been brought forth as another potential vaccine [110,111]. This vaccine also targets the CSP; however, in comparison to the RTS, S/AS01 vaccine, the difference is found in antigenic composition and adjuvant formulation. Clinical trials for this potential vaccine have reported 77% efficacy over 12 months in children aged 5–17 months, with increased immunogenicity and the potential for broad-spectrum implementation [112]. Together, RTS, S, and R21 are promising therapeutics for malaria prevention and decreasing rates of incidence. A third type of vaccine, sexual-stage vaccines, can stop the sexual development of the parasite in the *Anopheles* spp. mosquito; however, blocking the parasite transmission has had limited progress because of insufficient funding [113].Novel approaches, including mRNA platforms and genetically attenuated parasites, and multi-stage vaccines, are also under investigation [114]. Key challenges remain, such as ensuring vaccine affordability, addressing supply-chain complexities, and overcoming the antigenic diversity of malaria parasites.

#### 3.2.2. Monoclonal Antibodies and Immune Modulators as Potential Therapies

Monoclonal antibodies are novel interventions against malaria as a means of a prophylactic and therapeutic regimen by targeting specific antigens. PfRH5 (reticulocyte-binding protein homolog 5) is a protein surface marker for erythrocyte invasion; antibodies have been studied to inhibit parasite replication [115]. There have been in-depth studies of immunomodulators as adjunctive therapy for infection. Effective and significant results have been shown in animal studies; however, translational results have not been witnessed in human studies [116]. These immunotherapies are in the initial stages of being used as potential therapy strategies to reduce parasite burden and improve vaccine efficacy, especially in drug-resistant malaria [117].

#### 3.2.3. Combination Therapies

Combination therapies play a critical role in modern malaria treatment strategies, particularly in combating drug-resistant strains of the parasite. Artemisinin-based combination therapies (ACTs) pair a fast-acting artemisinin derivative with a longer-acting partner drug such as lumefantrine, mefloquine, or piperaquine [118]. This dual approach allows for a rapid reduction in parasite biomass by artemisinin, while the partner drug clears any remaining parasites, significantly lowering the risk of resistance to either component.

To address the growing threat of artemisinin-resistant *Plasmodium falciparum*, particularly in Southeast Asia and Eastern Africa, researchers have developed Triple ACTs (TACTs). These regimens combine artemisinin with two longer-acting drugs—for example, artemisinin–piperaquine–mefloquine or artemisinin–amodiaquine–lumefantrine [119]. TACTs offer broad-spectrum pharmacological coverage, increasing efficacy and reducing the likelihood of treatment failure in regions with multidrug resistance.

In addition to targeting the parasite directly, adjunctive therapies are being explored to address host-related complications of malaria, such as oxidative stress, inflammation, and endothelial damage. Preclinical studies have shown that antioxidants, like vitamin C and E, can reduce reactive oxygen species and impair parasite growth within the host [120]. Iron chelators, such as deferoxamine, further contribute by sequestering iron, which is essential for parasite metabolism, while simultaneously mitigating oxidative stress in the host [121].

Beyond antioxidants, hormonal and metabolic modulators are also gaining attention. For instance, the PPARγ (peroxisome proliferator-activated receptor gamma) agonist rosiglitazone has shown promise in both preclinical and clinical settings. It appears to reduce inflammation and endothelial injury while enhancing parasite clearance in malaria-infected individuals [122]. These adjunctive and combination therapies illustrate a multi-targeted approach to malaria treatment, aiming not only to eliminate the parasite but also to manage the host’s physiological response for improved outcomes.

#### 3.2.4. The Role of Combination Drug Regimens to Overcome Resistance

Combination therapies assist in treatment efficacy and have been shown to delay the development of antimalarial drug resistance [123]. Employing this form of drug regimen exposes the parasite to various drugs and their respective mechanisms of action. As a result, this reduces the probability of the parasite developing resistance to many of the drugs involved in the regimen [119]. Resistance to chloroquine appeared rapidly when used as monotherapy, whereas ACTs have sustained efficacy for nearly two decades due to their dual-drug format [124]. Studies have demonstrated that using combination regimens can extend the effective lifespan of existing antimalarials and reduce the frequency of treatment failures [125].

Emerging strategies include combining new drug candidates, like cipargamin (a PfATP4 inhibitor) or ganaplacide (a PI4K inhibitor, phosphatidylinositol 4-kinase), with existing therapies to improve efficacy and delay resistance emergence [85]. These novel combinations are being evaluated in clinical trials, with early data suggesting promising activity against both asexual blood stages and gametocytes [126]. Such combinations may also allow for single-dose treatments, improving patient adherence and public health outcomes. Continued surveillance of resistance markers and real-world efficacy studies will be vital to guide the optimal dosage of combination regimens.

#### 3.2.5. Synergistic Effects of Existing Drugs with Novel Agents

Drug synergy is the effect where the sum of the drugs exceeds the sum of their individual effects. Drug combinations are also being studied to target and disrupt the various stages of the *Plasmodium* life cycle [127]. Implementing synergistic combinations reduces overall toxicity, as the required dose of each component is decreased. Studies have shown some promise using a combination of artemisinin with atovaquone, a mitochondrial electron transport blocker, to effectively eliminate the parasite, including artemisinin-resistant species [128]. The synergistic effect of chloroquine and tafenoquine—a transmission-blocking agent—has shown a reduction in clinical symptoms and an inhibition of gametocyte transmission to mosquitoes [129].

#### 3.2.6. Molecular Markers for Diagnostics to Monitor Drug Efficacy and Resistance

Compared to traditional microscopy, molecular markers are instrumental for diagnostic purposes (especially in asymptomatic patients), monitoring drug resistance patterns, assessing elimination programs, and maintaining drug efficacy. PfEMP1 is a species-specific marker that provides an accurate diagnosis for *P. falciparum* [130], and, subsequently, allows for the proper treatment plan to be prescribed. Additionally, markers of resistance, such as pfcrt and pfmdr1 [131] for chloroquine and mefloquine resistance, dhfr (dihydrofolate reductase) and dhps (dihydropteroate synthase) for sulfadoxine-pyrimethamine resistance [132], and Kelch13 (K13) for artemisinin resistance [133], reveal information about necessary treatment guidelines as well as the spread of resistant infection. Monitoring these molecular markers allows for the localization of parasite populations and the identification of resistance patterns.

## 4. Challenges and Future Directions

### 4.1. Antimalarial Drug Resistance: Current Threats and Advances in Treatment

The emergence of drug-resistant *Plasmodium falciparum* strains represents one of the most pressing challenges in malaria control. Artemisinin partial resistance, linked to mutations in the Kelch13 gene, has now been detected in Africa among children with severe malaria, raising concerns about treatment efficacy in high-burden regions [134]. This resistance is exacerbated by the parasite’s ability to downregulate transfer ribonucleic acid (tRNA) modifications, which enhances survival under artemisinin pressure by altering stress-response pathways [135]. Concurrently, partner drug resistance (i.e., lumefantrine) further threatens artemisinin-based combination therapies (ACTs), as evidenced by post-treatment parasite recurrence in clinical studies [134]. To address this, novel non-artemisinin agents, including ganaplacide, a PI4K inhibitor, and reformulated lumefantrine, have demonstrated 95% efficacy in Phase 2b trials, offering a promising alternative to ACTs [136]. However, the prolonged half-life of legacy drugs, like chloroquine and mefloquine, continues to exert selective pressure, facilitating resistance spread through persistent subtherapeutic drug levels [137]. The strategic diversification of therapies, including multiple first-line drug combinations, could delay resistance by reducing the parasite’s exposure to uniform selection pressures. Accelerating the development of compounds targeting novel pathways (i.e., PfATP4 inhibitors, proteasome blockers) remains critical to outpace evolutionary adaptations in the *Plasmodium* spp.

### 4.2. Genetic and Molecular Mechanisms Driving Antimalarial Resistance in Plasmodium falciparum

The evolving nature of antimalarial resistance is driven by *Plasmodium falciparum*’s remarkable genetic plasticity, as demonstrated by systematic in vitro evolution studies revealing over 128 genes with mutations conferring resistance to 118 compounds [138]. Key resistance mechanisms include copy number variants (CNVs) amplifying drug targets or efflux pumps, including PfMDR1, and missense mutations in conserved protein domains, such as PfCRT and PfATP4, that alter drug binding or parasite physiology [138]. For instance, PfMDR1 amplifications reduce lumefantrine susceptibility by enhancing drug efflux, while Kelch13 mutations (i.e., C580Y, R561H) destabilize the propeller domain’s structure, enabling artemisinin tolerance through altered stress-response pathways [139,140]. These adaptations are compounded further by mutations in the AP2 transcription factor, which may coordinate multidrug resistance networks by regulating parasite stress adaptation [138]. To counter this, genomic surveillance platforms leveraging next-generation sequencing (NGS) now track emerging PfK13 and PfCRT variants across endemic regions, enabling dynamic adjustments to treatment policies [139,141]. The World Health Organization’s (WHO’s) Mekong Malaria Elimination program uses real-time resistance data to deploy ACTs (TACTs) in southeast Asia, combining artemisinin with two partner drugs to reduce resistance selection pressure [142]. Similarly, targeting non-mutable pathways, including PfATP4 ion homeostasis with cipargamin, exploits essential parasite biology with a lower resistance risk profile [138]. However, the continued use of established drugs, including chloroquine, continues to select resistant strains, emphasizing the need for coordinated drug withdrawal policies and a change to next-generation therapies with shorter half-lives [87].

### 4.3. The Importance of Continued Drug Discovery and Surveillance

The relentless evolution of drug-resistant *Plasmodium falciparum* necessitates sustained investment in novel antimalarial development and robust surveillance systems for resistance. Recent advancements include MED6-189 (a kalihinol analog), a dual-action compound targeting the apicoplast and vesicular trafficking pathways, which demonstrates efficacy against both drug-resistant and sensitive strains in vitro and humanized mouse models, with no observed emergence of resistance during trials [143]. Concurrently, genomic surveillance programs, including the Centers for Disease Control and Prevention’s (CDC’s) Malaria Resistance Surveillance (MaRS) project, employ advanced molecular detection (AMD) tools to identify resistance markers (i.e., Kelch13, PfCRT) in real time to enable rapid policy adjustments in regions including the Greater Mekong Subregion (GMS) [119,144]. For example, Vietnam and Laos have revised front-line therapies based on genomic data and therapeutic efficacy studies (TES), resulting in a 30% reduction in treatment failures in high-transmission zones [144]. Despite progress, funding gaps persist, as only 18% of endemic countries have integrated genomic surveillance into their national programs and preclinical pipelines for non-artemisinin agents remain under-resourced [145]. Public–private partnerships, including the Medicines for Malaria Venture (MMV), are crucial for accelerating the development of treatments, such as cipargamin (a PfATP4 inhibitor) and ganaplacide (a PI4K inhibitor), through clinical stages [84,145]. Harmonizing these efforts with climate-resilient surveillance frameworks and equitable funding distribution is pivotal to countering multidrug-resistant strains and achieving elimination targets.

### 4.4. From Drug Resistance to Vaccine Breakthroughs: Advances in Malaria Control

The history of malaria treatment has been marked by a relentless cycle of drug development and resistance. Chloroquine, introduced in the 1940s, initially reduced global malaria mortality by 95% until resistance linked to PfCRT mutations emerged in southeast Asia by 1957 and spread globally, rendering it ineffective in most regions by the 1990s [146]. Sulfadoxine-pyrimethamine (SP), adopted as a replacement to chloroquine, experienced resistance linked to dhfr/dhps mutations, with treatment failure rates exceeding 50% in Africa by the early 2000s [147]. These setbacks signified the parasite’s ability to evade single-target therapies through genetic plasticity and selection pressure from prolonged drug use. The shift to artemisinin-based combination therapies (ACTs) in the 2000s aimed to delay resistance; however, partial artemisinin resistance (Kelch13 mutations) emerged in southeast Asia and recently in Africa, reducing parasite clearance by 50% in some regions [148]. This has influenced global initiatives for innovative strategies, paving the way for the development of vaccines.

Modern vaccine efforts have achieved milestones with the development of the RTS,S/AS01, and R21/Matrix-M vaccines. Developed through a decades-long partnership between GlaxoSmithKline (GSK), the University of Oxford, PATH Malaria Vaccine Initiative (MVI), and African research institutions, both vaccines represent innovative breakthroughs by inducing immune responses against the *Plasmodium falciparum* circumsporozoite protein (CSP) to prevent sporozoites from infecting the liver. Developed by GSK, the RTS,S/AS01 combines CSP fragments with hepatitis B surface antigen (HBsAg) to form virus-like particles, adjuvanted with AS01 to enhance immunogenicity [149,150]. Phase 3 trials demonstrated 36% efficacy over four years in children aged 5–17 months, leading to the WHO’s recommendation in 2021, after pilot implementations in Ghana, Kenya, and Malawi showed a 13% reduction in mortality [109,151].

The R21/Matrix-M vaccine is a novel vaccine that utilizes a higher CSP-to-HbsAg ratio and the Matrix-M adjuvant, achieving 65–77% efficacy in Phase 3 trials by enhancing anti-CSP antibody titers [152,153]. The WHO endorsed R21 in 2023, citing its cost-effectiveness and scalability, with a production capacity of 200 million annual doses [154,155]. While RTS,S laid the foundation for malaria vaccine development, R21’s higher efficacy and manufacturability address gaps in global supply, with both vaccines now integrated into the WHO’s strategy to reduce malaria morbidity and mortality in endemic regions. These vaccines represent significant progress; however, their modest efficacy and logistical challenges reflect the broader complexities of malaria immunology.

### 4.5. Challenges in Creating an Effective Malaria Vaccine

Developing a highly efficacious malaria vaccine faces biological and logistical barriers, primarily due to the antigenic variation, genetic diversity, and multi-stage life cycle of *P. falciparum*. The var gene-coded PfEMP1 family exemplifies the parasite’s antigenic variation, as its hypervariability enables immune evasion through cytoadhering and antigenic switching, undermining strain-transcendent immunity [148,156]. While RTS,S/AS01, and R21/Matrix-M target conserved CSP regions, their efficacy (29–77%) and waning protection highlight limitations in addressing antigenic diversity and liver-stage persistence [110,157]. Transcriptomic studies reveal that protected individuals exhibit early interferon and natural killer (NK) cell responses following challenge; however, these correlates lack consistency across populations, complicating vaccine design [158]. Furthermore, the parasite’s multi-stage life cycle necessitates targeting both pre-erythrocytic (sporozoite) and blood-stage (merozoite) antigens; few candidates (i.e., PfRH5, Pfs25) have demonstrated cross-stage efficacy in clinical trials [159,160]. Blood-stage candidates, such as PfRH5, show 40–55% efficacy in Phase 2B trials against blood-stage parasites with broad neutralizing potential [161,162]. Concurrently, host immune exhaustion from recurrent infections may synergize with antigenic diversity to limit vaccine durability.

Recent genomic surveillance emphasizes the urgency of *P. falciparum* resistance to antimalarial drugs and highlights the need for accelerated research into new treatments and interventions. Populations in Africa exhibit a 12–15% divergence in CSP sequences, potentially reducing vaccine efficacy in highly transmissible regions [163]. Genomic surveillance confirms *P. falciparum’s* resistance to artemisinin derivatives through AP2 transcription factors, potentially undermining chemoprophylaxis-dependent vaccine strategies [138,164,165,166]. This pharmacological failure may exacerbate immunosuppression by permitting chronic parasite exposure.

Beyond genetic diversity and pharmacological challenges, host immunological barriers, particularly immunosuppression from prior exposure, further compromise vaccine efficacy. Parasite-derived immunosuppressive factors (i.e., PfEMP1 and PfTRAP [*Plasmodium falciparum* thrombospondin-related anonymous protein]) and cytokines (IL-10 [interleukin-10], TGF-β [transforming growth factor beta]) induce endotoxin-like tolerance, impairing dendritic cell maturation and promoting regulatory T-cell expansion through TGF-β activation [167,168]. Elevated immunosuppressive cytokines (IL-10, TGF-β) blunt antibody production and Th1 (T helper type 1 cells) responses, creating a milieu that impedes vaccine-induced immunity [169,170]. Malaria-associated chemokines (CCL17 [C-C motif chemokine ligand 17], CCL22) exacerbate this suppression by promoting regulatory dendritic cell phenotypes that inhibit effector T-cell priming and the recruitment of immunosuppressive cells, while elevated CXCL11 (C-X-C motif chemokine ligand 11) and CCL28 correlate with impaired vaccine efficacy and adverse clinical outcomes in endemic populations [148,169,170,171,172,173]. Clinically, this manifests as reduced efficacy; RTS,S/AS01 achieves higher protection in naïve individuals than those with ongoing malaria or prior exposure, and endemic populations exhibit lower antibody titers due to pre-existing immunosuppressive microenvironments [174,175].

### 4.6. Immunological Hurdles and the Need for Novel Vaccine Platforms

The immunological barriers to developing a malaria vaccine are significant. As stated previously, the var gene-coded PfEMP1 family enables the parasite to evade immune detection through rapid antigenic switching, making it difficult for vaccines to induce protection. Immune correlates, such as early interferon and NK cell responses, vary between individuals and populations, complicating the rational design of vaccines. To overcome these hurdles, novel vaccine platforms are being developed to induce durable cross-protective immunity [164,165,176].

Current platforms, including RTS,S/AS01, and R21/Matrix-M, targeting the parasite’s CSP, face critical limitations. While RTS,S induces anti-CSP antibodies and CD4+ T-cell responses, its efficacy wanes rapidly (39% over four years), partly due to antibody titers declining post-immunization, and to strain-specific immunity against heterologous CSP variants [150,177]. Similarly, R21’s 77% efficacy in Phase 2b trials diminishes after 12–18 months, reflecting the poor durability of humoral responses and inadequate T-cell memory [150,178]. Both vaccines struggle against Africa’s genetically diverse *P. falciparum* populations, where CSP sequences diverge by 12–15%, enabling vaccine escape [150,179]. The immunity correlates with protection, including early interferon-γ and NK cell activation, which vary significantly between individuals and populations, complicating the rational design of vaccines [158,180]. For instance, transcriptomic analyses reveal that protected vaccines exhibit distinct type I/II interferon signatures post challenge, while non-protected individuals show dysregulated innate and adaptive responses [158].

Novel platforms aim to overcome these hurdles. mRNA-containing lipid nanoparticle (LNP) vaccines encoding CSP or transmission-blocking antigens induce robust, cross-reactive antibodies and CD8+ T-cell responses in preclinical models, outperforming traditional subunit vaccines [181]. Similarly, monoclonal antibodies, including CIS43LS, which targets CSP, achieved 88% efficacy in Phase 2 trials by neutralizing sporozoites within minutes of mosquito inoculation [182]. Platforms combining multi-stage antigens (i.e., PfCSP + Pfs25 mRNA-LNPs) or targeting immune evasion factors (i.e., *Plasmodium* macrophage migration inhibitory factor [PMIF] via self-amplifying RNA) show promise in eliciting durable, cross-protective immunity [150,181]. However, challenges have persisted. Correlates of protection remain elusive, with systems serology highlighting the importance of Fc-mediated effector functions (i.e., antibody-dependent cellular phagocytosis) over antibody titers [183]. Preimmunization host factors, including baseline inflammatory states, influence vaccine responsiveness, suggesting that personalized approaches may be needed [158,180].

### 4.7. Global Health and Equity Considerations

Malaria continues to be a disease intricately linked to poverty, disproportionately impacting the world’s most impoverished populations and exacerbating existing health inequalities, with cases and deaths remaining overwhelmingly concentrated in the WHO African region, where many people at risk still lack access to essential prevention, diagnosis, and treatment services [184,185,186]. Socioeconomic status (SES) profoundly shapes malaria risk and outcomes: individuals with lower income, limited education, and inadequate housing are more likely to be exposed to malaria vectors and less likely to access effective interventions [187,188,189]. Studies have consistently shown that wealthier households are 1.25–2.5 times more likely to receive key public services, including malaria interventions, compared to poorer households [187,190]. Catastrophic financial burdens are common. For example, in Nigeria, malaria treatment costs consume 7.8% of monthly non-food household expenditures for the poorest quintile, compared to 3.9% for wealthier households [187]. Gender disparities further exacerbate the issue, with pregnant women often lacking access to safe therapies due to exclusion from clinical trials and limited healthcare autonomy in patriarchal societies [187]. Only 12% of antimalarial trials include pregnant women, leaving critical gaps in evidence-based care [187]. Rural and marginalized populations, including children under five years old, migrants, and Indigenous peoples, are especially vulnerable due to geographic isolation, under-resourced health infrastructure, and reliance on unqualified providers or counterfeit drugs [184,191]. Climate change intensifies these challenges by expanding Anopheles mosquito habitats into new regions, disproportionately affecting communities with limited adaptive capacity [192].

#### 4.7.1. Access to New Therapies in Low-Income Regions

Access to new therapies in low-income regions remains a difficult task. Systemic barriers, such as high costs and weak infrastructure, have hindered the widespread use of artemisinin-based combination therapy (ACT) alongside vaccines. ACTs are often priced three times higher than older therapies, like chloroquine, making them inaccessible for many households in sub-Saharan Africa and other malaria-endemic regions [193]. Additionally, in high-burden countries, such as Nigeria and Tanzania, 60–80% of antimalarial prescriptions are dispensed by unqualified providers, frequently resulting in substandard or incomplete treatment, thereby perpetuating drug resistance and poor health outcomes [193]. The rollout of malaria vaccines faces similar obstacles: only about 35% of health facilities in sub-Saharan Africa have reliable cold-chain capacity, a critical requirement for storing and distributing both RTS,S/AS01, and R21/Matrix-M, which are highly temperature-sensitive [194,195]. Multi-dose regimens further complicate adherence, particularly in rural and remote areas with limited healthcare access [196,197]. Community health worker (CHW) programs and subsidized drug initiatives have improved access in some pilot settings, reducing malaria mortality and expanding access to underserved populations [196]. However, scaling these efforts remains difficult due to chronic underfunding. Annual investments of USD 6.8–7.7 billion are required to achieve global malaria targets [193]. However, funding falls short by nearly 50%, with low-income countries receiving only a fraction of the needed resources [193,196]. These barriers underscore the urgent need for innovative financing, tiered pricing, regional manufacturing partnerships, and robust international support to ensure that life-saving therapies and vaccines reach those most in need and to close the equity gap in malaria control [193,196].

#### 4.7.2. Strategies for Equitable Distribution of Antimalarial Treatments and Vaccines

Equitable access to malaria interventions requires multi-layered strategies addressing systemic barriers in resource-limited settings. The WHO’s prioritized allocation framework, which directs vaccines, such as RTS,S/AS01, and R21/Matrix-M, to high-transmission districts, has reduced disparities by ensuring that 90% of children in pilot countries benefit from at least one preventive intervention, including ITNs or vaccines [175,198]. However, fragile health infrastructure, including inconsistent cold-chain capacity and gaps in healthcare worker training, remains a critical bottleneck, with only 35% of facilities in sub-Saharan Africa equipped to store temperature-sensitive vaccines [199]. To mitigate this, initiatives, like the AMVIRA program, deploy dashboards to track district-level readiness and vaccine uptake while training community health workers (CHWs) to deliver doses in remote areas [200]. Equitable distribution also requires that financial barriers be addressed. Tiered pricing models, subsidized through Gavi’s funding targets, have lowered R21 costs to USD 3–4 per dose for low-income countries, although counterfeit drugs still dominate 20% of the markets in underserved regions [201]. Integrating vaccines with existing tools, such as layering RTS, S/AS01 with seasonal malaria chemoprevention (SMC) in Sahelian Africa, has boosted protection rates to 75% in children under five [202]. Real-time genomic surveillance of resistance markers (i.e., pfk13, pfcrt) further enables the dynamic reallocation of therapies to hotspots, while community-led demand campaigns, such as Benin’s storytelling-driven rollout, counter misinformation and improve uptake by 40% [199]. Sustained progress hinges on decentralizing manufacturing through partnerships, such as the Serum Institute’s African production hubs, and enforcing stricter penalties for counterfeit drug networks [201].

#### 4.7.3. The Role of Collaborative Global Initiatives

**Figure 3:**
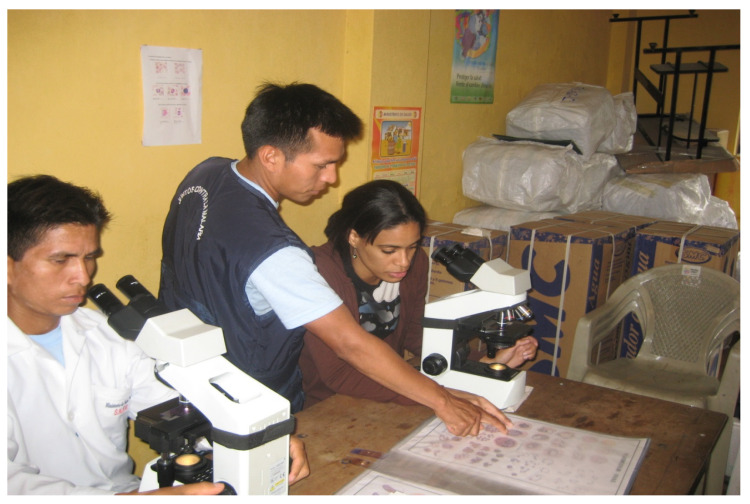
Malaria timeliness diagnosis and treatment carried out by Ecuadorian Amazonian rainforest shamans and USA public health students (courtesy of Dr Izurieta Malaria Seek and Destroy Team).

Collaborative global initiatives have proven instrumental in advancing malaria control by fostering cross-border coordination and resource optimization. Regional partnerships, such as the MOSASWA initiative (Mozambique, South Africa, and Eswatini), demonstrate the impact of joint interventions, including synchronized insecticide-treated net distribution and surveillance systems, which have reduced malaria incidence by 45% in border regions between 2015 and 2022 [203]. Similarly, the Asia Pacific Malaria Elimination Network (APMEN) utilizes data-sharing platforms to monitor drug-resistant strains across 14 countries, facilitating preemptive treatment adjustments in high-risk areas [203]. The E8 Initiative (Elimination 8 Regional Initiative), uniting eight southern African nations, combines pooled procurement of diagnostics with cross-border rapid-response teams, achieving a 60% decline in malaria-related deaths in Zambia and Zimbabwe since 2020 [204]. These efforts are strengthened by frameworks, including the WHO’s Global Technical Strategy 2016–2030, which prioritizes regional capacity-building and standardized protocols for vector control and case management such as the Seek and Destroy Strategy to Eliminate Malaria (Figure 3) [193].

However, challenges persist. Initiatives, including the Trans-Kunene Malaria Initiative (Namibia-Angola), rely on volatile donor funding for 80% of their budgets, risking program continuity during economic downturns [203]. Despite this, collaborative models remain critical for addressing mobile populations and the genetic diversity of parasites, with the Roll Back Malaria (RBM) Partnership reporting a 33% faster decline in malaria mortality in countries engaged in multinational coalitions compared to those acting unilaterally [204].

#### 4.7.4. Role of Organizations Like the WHO, Gates Foundation, and Other Entities in Combating Malaria

Organizations, including the WHO, the Gates Foundation, and the Global Fund, drive malaria eradication through targeted funding, research, and policy leadership. The WHO’s Global Malaria Program coordinates surveillance networks and treatment guidelines, recently deploying real-time resistance monitoring systems in 15 African nations to track pfk13 and pfcrt mutations [205]. The Gates Foundation has committed over USD 4 billion since 2000, catalyzing breakthroughs, like the RTS and S/AS01, vaccine and supporting the Medicines for Malaria Venture (MMV), which has advanced 15 novel compounds to clinical trials since 2015 [206,207]. Meanwhile, the President’s Malaria Initiative (PMI), a joint effort between the CDC and United States Agency for International Development (USAID), has distributed 800 million insecticide-treated nets and trained 250,000 healthcare workers across sub-Saharan Africa since 2005, contributing to a 44% regional reduction in child mortality [208]. Private sector alliances, such as ExxonMobil’s Malaria Initiative, partner with endemic countries to strengthen supply chains, delivering 15 million rapid diagnostic tests to Nigeria and Angola between 2018 and 2023 [209,210]. These entities also address systemic inequities: the Global Fund allocates 65% of its malaria funding to low-income nations, while the RBM Partnership advocates for tiered pricing to ensure that therapies, like artemether-lumefantrine, remain accessible at USD 0.50 per dose in high-burden areas [211]. Together, these organizations form an interconnected ecosystem that combines innovation, equity, and scalability to counter evolving malaria threats.

#### 4.7.5. The Importance of Funding and International Collaboration for Sustained Progress

Sustained progress in malaria control and elimination hinges on robust funding and international collaboration, which underpin the effectiveness of global and regional initiatives. The dramatic decline in malaria incidence and mortality over the past two decades has been attributed primarily to increased international financing and the coordinated efforts of multilateral partnerships, such as the Global Fund, which currently provides over 60% of global malaria funding and has invested more than USD 19 billion in control programs [212,213]. This influx of resources has enabled the scaling of proven interventions, including insecticide-treated nets, diagnostics, and new vaccines, across diverse and hard-to-reach populations [213]. Recent stagnation in financial support, coupled with an annual shortfall of USD 4.3 billion, now threatens to undermine previous progress made, particularly as rising drug resistance and the continued spread of disease across borders require even more flexible operations and increased resource sharing [203]. To maintain the viability of these initiatives, adopting innovative financial approaches and building partnerships with philanthropic organizations are vital to traditional aid mechanisms [203]. Working together across countries bolsters international commitments by exchanging epidemiological information to improve further strategies for addressing issues, including imported malaria and controlling the spread of resistance [203,214]. A fully funded and globally coordinated response is vital to maintaining momentum, bridging equity gaps, and achieving the ambitious 2030 malaria elimination targets.

## 5. Conclusions

Malaria remains a major global health burden, driven by the intricate biology of the *Plasmodium* parasite and compounded by persistent social and health inequities. This paper has explored the molecular mechanisms that underpin malaria pathogenesis, including red blood cell invasion, immune evasion through antigenic variation, and the emergence of drug resistance. It has also reviewed promising therapeutic advances such as novel antimalarial agents, monoclonal antibodies, and multi-stage vaccine platforms.

While these innovations mark important progress, the path to malaria eradication demands sustained research efforts. Future studies must focus on identifying non-mutable drug targets, improving vaccine durability and cross-strain protection, and developing strategies to block transmission at multiple stages of the parasite life cycle. Expanding genomic surveillance and integrating multi-omics approaches will be essential to track resistance trends and guide precision therapies.

Ultimately, malaria elimination will require a coordinated global effort—one that unites scientific discovery with equitable implementation. By investing in next-generation research and strengthening international collaboration, we can accelerate progress toward a malaria-free future.

## Data Availability

No new data were created or analyzed in this study. Data sharing does not apply to this article.

## Abbreviations

The following abbreviations are used in this manuscript:

PfCRT	*Plasmodium falciparum* chloroquine resistance transporter
PfMDR1	*Plasmodium falciparum* multidrug resistance protein 1
DHODH	Dihydroorotate dehydrogenase
ITNs	Insecticide-treated bed nets
RDTs	Rapid Diagnostic Tests
WHO	World Health Organization
CDC	Centers for Disease Control and Prevention’s
ACT	Artemisinin-based combination therapy
RBM	Roll Back Malaria
USAID	United States Agency for International Development
APMEN	Asia Pacific Malaria Elimination Network
G6PD	Glucose-6-phosphate dehydrogenase
